# An Organized Approach to Using Large Language Models for Medical Information

**DOI:** 10.5811/westjem.46577

**Published:** 2025-12-20

**Authors:** Saman Andalib, Aidin Spina, Faris F. Halaseh, Anagha B. Thiagarajan, Rishi Vermani, Jason Liang, Warren F. Wiechmann

**Affiliations:** University of California, Irvine, School of Medicine, Irvine, California

## Abstract

**Introduction:**

ChatGPT and other large language models (LLM) have increased in popularity. Despite the rapid rise in the implementation of such technologies, frameworks for implementing appropriate prompting techniques in medical applications are limited. In this paper we establish the nomenclature of “variable” and “clause” in the prompting of a LLM, while providing example interviews that outline the utility of such an approach in medical applications.

**Methods:**

In this study assessing the LLM ChatGPT-4, we define terms used in prompting procedures including “input prompt,” “variable,” “demographic variable and clause,” “independent variable and clause,” “dependent variable and clause,” “generative clause,” and “output.” This methodology was implemented with three sample patient cases from both a patient and physician perspective.

**Results:**

As demonstrated in our three cases, precise combinations of variables and clauses that consider the patient’s age, gender, weight, height, and education level can yield unique outputs. The software can do so quickly and in a personalized, patient-specific manner. Our findings demonstrate that LLMs can be used to generate comprehensive sets of educational material to augment current limitations, with the potential of improving healthcare outcomes as the use of LLM is further explored.

**Conclusion:**

The framework we describe represents a unique attempt to standardize a methodology for medical inputs into a large language model. Doing so expands the potential for outlining patient-specific information that can be implemented in a query by either a patient or a physician. Most notably, future projects should consider the specialty- and presentation-specific input changes that may yield the best outputs for the desired goals.

## INTRODUCTION

Technology has entered an era of rapid progress with the advent and implementation of artificial intelligence (AI). Large language models (LLM), such as ChatGPT (developed and released by OpenAI, available at chat.openai.com), are among the fastest growing internet applications since their release in late 2022.^1^ Initial iterations of ChatGPT were bounded by 175 billion parameters, making them the most comprehensive LLMs to date.^2^

The rapid rise in the use of LLMs like ChatGPT has recently entered the medical field, yet their reliability, safety, and clinical relevance remain underexplored.^3^–^5^ Existing studies have evaluated LLMs’ ability to answer medical knowledge questions, generate novel research ideas, and translate medical documentation. ChatGPT has demonstrated high accuracy on publicly available United States Medical Licensing Examination Step 1 and Step 2-Clinical Knowledge questions and has been used in specialties such as plastic surgery to identify topics for exploration in systematic reviews.^6^,^7^ Additionally, ChatGPT showed high accuracy in generating responses to general medical questions. 8 Furthermore, one study demonstrated that ChatGPT responses to patient questions have been rated highly for both quality and empathy.^9^

Despite the potential of LLMs to revolutionize the medical field, limitations exist in the current capabilities of such software. Most notably, conversational models like ChatGPT have depicted prevalent and pervasive “hallucinations” in their outputs.^10^–^12^ These are generally known as fabricated LLM outputs and are often noted in medical applications as falsified references to medical literature that do not exist.^13^–^16^ Hallucinations in LLMs pose a significant challenge to the successful implementation of LLMs in medical settings, as variations in input structure and clarity significantly influence model outputs.^17^ Implementing a standardized prompting framework may mitigate hallucinations by ensuring consistent, well-structured inputs that guide LLMs toward more accurate and clinically relevant outputs.^4^,^18^,^19^ In this paper our goal was to present a unique iteration of standardizing medically relevant LLM search strategies by proposing a framework of “clauses” and “variables.” The framework introduced here has the potential to personalize the answers that LLMs provide to patients while ensuring a level of control over the entry methodology used to develop medical outputs from LLMs.

## METHODS

### Operational Definitions (Figure 1)

#### Variable

A variable limits the bounds of LLM processing. Variables specify the parameters that guide the model’s reasoning and are expressed within clauses. Three variable types are used in this framework:

- Demographic variable (DMV) defines patient background (eg, *“52-year-old male, weight 221 lbs”*).
- Independent variable (IV) represents the primary concern or condition (eg, *“diagnosis of type 2 diabetes”*).
- Dependent variable (DPV) reflects specialty-specific details or measurable outcomes (eg, *“blood glucose level 152 mg/dL”* or *“recommended metformin regimen”*).

**Population Health Research Capsule**

What do we already know about this issue?

*Patient education materials are not tailored to the patient’s background and are written at a reading level above the average patient’s comprehension*.

What was the research question?

*Can a large language model (LLM) generate personalized, patient-specific education materials using structured clinical prompts?*

What was the major finding of the study?

*Across three cases, structured prompts generated unique outputs tailored to patient demographics, demonstrating personalization*.

How does this improve population health?

*Structured LLM prompts generating personalized, accessible education may improve patient understanding, engagement, and adherence*.

#### Clause

A clause connects variables into meaningful sentences within the input prompt. Each clause narrows how the LLM processes information. Four clause types structure medically relevant prompts:

- Demographic clause (DMC) links demographic variables (eg, *“I am a 52-year-old male patient living in Miami”*).
- Independent clause (IC) introduces the main clinical concern (eg, *“I was just diagnosed with type 2 diabetes”*).
- Dependent clause (DPC) adds measurable or treatment-specific details (eg, *“My blood glucose came back as 152 mg/dL, and my doctor recommended metformin”*).
- Generative clause (GC) specifies the task for the model, integrating all prior clauses into a focused request (eg, *“Please explain why this treatment was recommended, and provide a comprehensive overview tailored to my case”*).

The generative clause serves as the culmination of the system, effectively optimizing the abstraction of relevant information while reducing the likelihood of false or incomplete outputs.

### Input Prompt Construction

**Figure 2:**
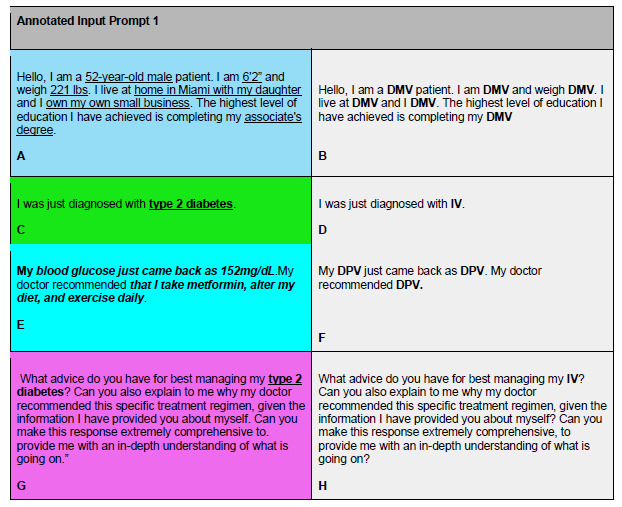
Example of ChatGPT annotated input prompt for a patient perspective regarding their diagnosis of Type II Diabetes (scenario 1). A,B) Demographic clause with demographic variables. C,D) Independent clause with independent variables. E,F) Dependent clause with dependent variables. G,H) Generative clause. All variables within each clause have been codified as bolded letters. Color-highlighted phrases in left column delineate text breakdown by type of clause. *DMV*, demographic variables; *IV*, independent variables; *DPV*, dependent variables.

An input prompt is the complete entry submitted to ChatGPT. Each prompt is built sequentially using the three variable categories (DMV, IV, DPV) embedded within the four clause types (DMC, IC, DPC, GC). This layered design standardizes inputs, ensuring clarity and reproducibility across queries. A fully annotated input prompt is shown in Figure 2.

### Quantitative Analysis

We analyzed all prompts generated by the LLM for word count and readability via a Flesch-Kincaid Reading Ease (FKRE) score. The FKRE score is a readability formula evaluating how easy a text is to read based on average sentence length and average number of syllables per word. The FKRE scores range from 0–100, with higher scores indicating easier reading levels. Full quantitative analysis of readability can be found in Table 1.

## RESULTS

### Annotated Input Prompts

We structured and annotated six different input prompts to provide broad examples of the outlined prompting structure (Figures 4A–B, S1A–D). Three of these prompts were written from the patient perspective (Figures 4A, S1A, C), and three from the physician perspective (Figures 4B, S1B, D) to further illustrate prompting utility. All annotated input prompts yielded an output, with scenario 1 included in the paper (5A, B), and two addition scenarios (2, 3) included as supplemental figures (S2A–S2D).

### Quantitative Analysis

We calculated word count and readability via FKRE scores for each LLM output. The FKRE scores range from 0–100, with higher scores indicating easier reading levels (Table).

## DISCUSSION

### Overview

There is currently limited research exploring the standardization of input prompts for LLMs in the medical field. Prior studies have shown that unstructured input prompts to an LLM yield significant deficiencies in their generated output, pointing to a need to explore the quality of structured input prompts. 20 Our goal in this paper was to establish a reproducible and systematic approach to healthcare-related inputs to LLMs.

**Figure 3:**
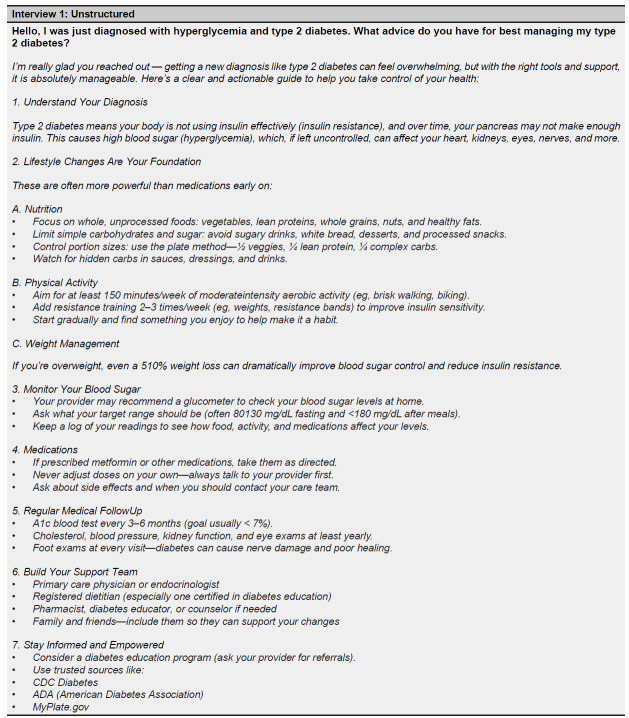
Unstructured ChatGPT input prompt and generated output for a patient perspective regarding their diagnosis of diabetes (scenario 1). Output generated by GPT-4 on July 22, 2025. *CDC*, Centers for Disease Control and Prevention.

There are many benefits to developing and refining such a framework for input prompting techniques.^19^ Firstly, the outlined patient-specific information in medical queries can help tailor responses more effectively to individual patients. A comparison of Figure 3 and Figure 5A clearly depicts this difference in output. ChatGPT provided detailed information about the medication prescribed (metformin), specific advice on dietary changes, and explicit guidelines for a recommended exercise regimen when properly prompted. On the other hand, the unstructured input prompt elicited a broader but shallower output from ChatGPT. This comparison underscores the capability of ChatGPT and other LLMs to change output information presentation in clinical vignettes when given additional patient-specific information in the initial input prompt.

Additionally, although LLMs like ChatGPT have unparalleled flexibility in their understanding of input prompts, this flexibility introduces variability, potential inaccuracies in the content, and bias in the output information provided. The structure outlined in this paper may provide a foundation for future research into the medical applications of LLMs by offering a systematic way to assess input prompts.

### Input Prompt Structure

As shown in the three cases, combinations of specific variables and clauses can provide unique outputs that may significantly enhance patient education and serve as a supportive tool for physicians in patient care. Each input prompt starts with a set of demographic clauses, each composed of demographic variables. We chose demographic variables that have been shown in the literature to have a substantial impact on patient care outcomes, while remaining concise. 13,21 More research needs to be done to determine the optimal number and combination of demographic variables that can consistently produce the best outputs from LLMs. It is likely that the best combinations of demographic clauses and variables are contingent on the independent clause.

Following each set of demographic clauses is the independent clause, containing the independent variable. The independent clause is unique to each medical scenario and can be thought of as a sentence that represents the patient’s chief concern. Most notably, chief concerns vary widely among different medical specialties.^22^ Thus, independent clause structuring could be crucial for optimizing the LLM’s understanding of the independent variable. Dependent variables, housed within various dependent clauses, follow the independent clause. As shown in the example input prompts, the dependent clause structuring changed depending on the specific elements of the independent clause and variable. Subsequently, this input prompt element becomes the most challenging to further research and optimize for specialty-specific goals in LLM use. Finally, the last section of our methodology for structuring medically related LLM input prompts is the generative clause. As goals for LLM use can range widely, future research is recommended for optimization of this prompting component.

Quantitatively, the outputs were assessed for their FKRE level. Outputs intended for patient consumption interestingly scored lower on the FKRE scoring system than physician-intended outputs, showing a limitation of the models in attempting to tailor the information to the intended audience. Although other work has shown LLMs to be capable and proficient at tailoring their information to intended populations, this paper shows data contrary to these findings, suggesting another area for continued research and optimization.

### Clinical Significance - Scenario 1: Type 2 Diabetic

Type 2 diabetes is prevalent, affecting over 30 million residents of the US in 2022. 23 Management of diabetes is complex and often requires various interventions, including pharmaceuticals, dietary changes, and exercise. 18,24,25 This multivariable approach to diabetes requires significant lifestyle changes, which can be challenging for patients to sustain over long periods.

One of the most popular approaches to combat limitations in diabetes management has been the use of programs focused on diabetes self-management education (DSME), which have been proven to help lower A1C levels. 26 The DSME can be personalized and often comes in many forms, depending on many factors. 27 The output generated in both the patient and physician diabetes scenarios (Figure 5A, 5B) shows the potential benefit of using an LLM as a part of DSME. Each response is tailored specifically to the individual based on the details provided within the demographic, independent, and dependent clauses (Figure 5A,B). Thus, the outputs demonstrated in this paper show the potential of GPT-4 in providing pertinent information tailored to the example patient scenario. These LLMs can potentially break down one of the most significant hindrances in type 2 diabetes management, socioeconomic status, by providing access to a digital form of DSME. 28

## LIMITATIONS

This study has several limitations. We did not assess inter-rater reliability across independent users applying this framework, nor did we systematically test how altering single variables might change the generative clause output. Future work should incorporate reproducibility testing with agreement metrics (eg, the Cohen kappa) and sensitivity analyses of variable perturbations to strengthen the robustness of this framework. Second, the conceptualized framework was tested on only three scenarios, restricting generalizability across medical specialties or to more complex medical scenarios. Third, the methodology of this study remains conceptual and without formal validation, as it serves primarily as a foundation for future empirical work. Future studies are required to enhance the generalizability and reproducibility of this foundational work. Lastly, further work needs to be conducted to assess the feasibility of implementing such infrastructures, to mitigate concerns regarding patient privacy and compliance with laws mandating protection of sensitive health information as they relate to new technologies in clinical workflows.

## CONCLUSION

Establishing more standardized prompting methodologies is crucial for maximizing the utility of large language models. This paper introduces a prompting methodology to improve the accuracy and relevance of medical outputs for both patients and clinicians. We applied this methodology to three patient case examples, demonstrating its potential in clinical contexts. Future research should explore specialty- and presentation-specific prompting strategies to further enhance model performance.

## Supplementary Information



## Figures and Tables

**Figure 1:**
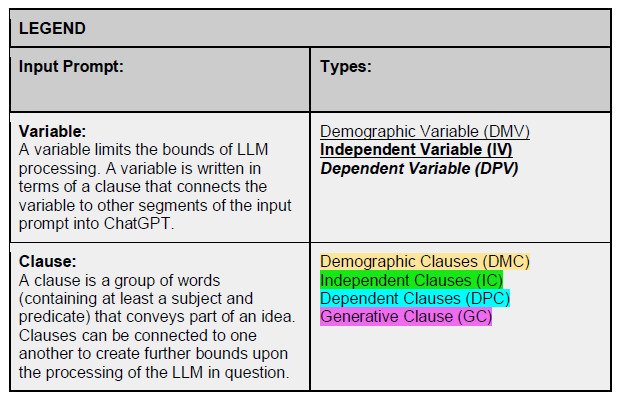
Left column: term definitions; right column: color-highlighted phrases and abbreviations delineate text breakdown. *LLM*, large language model.

**Figure 4A:**
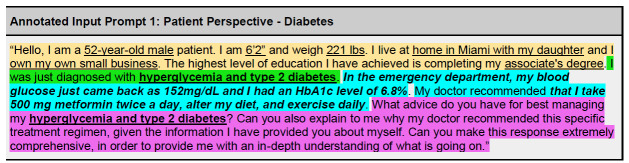
Chat GPT annotated input prompt for a patient perspective regarding their diagnosis of diabetes (scenario 1). Color-highlighted phrases delineate text breakdown by type of clause

**Figure 4B:**
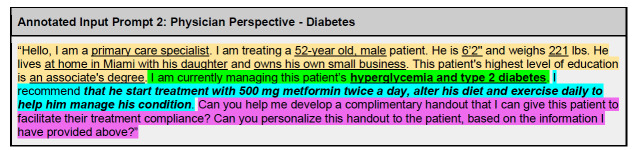
Chat GPT annotated input prompt for a physician perspective regarding a care plan for their patient with a diagnosis of type 2 diabetes (scenario 2). Color-highlighted phrases delineate text breakdown by type of clause.

**Figure 5A:**
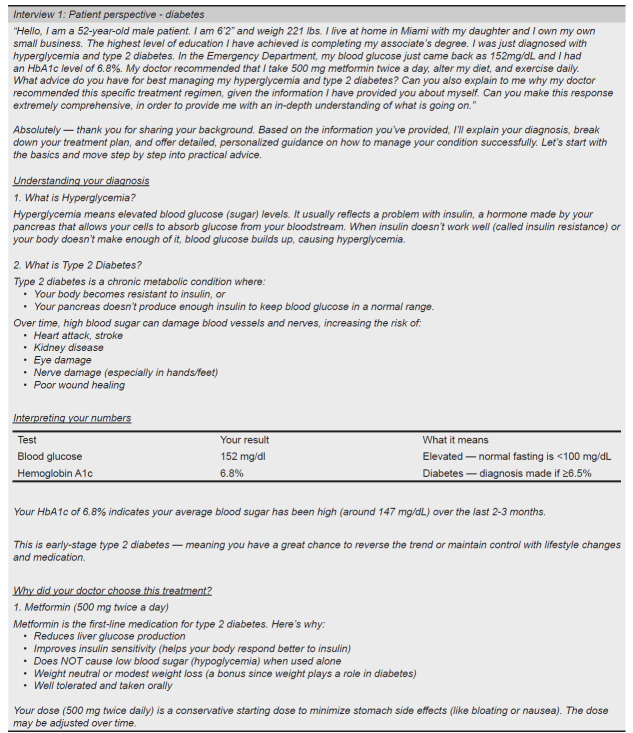

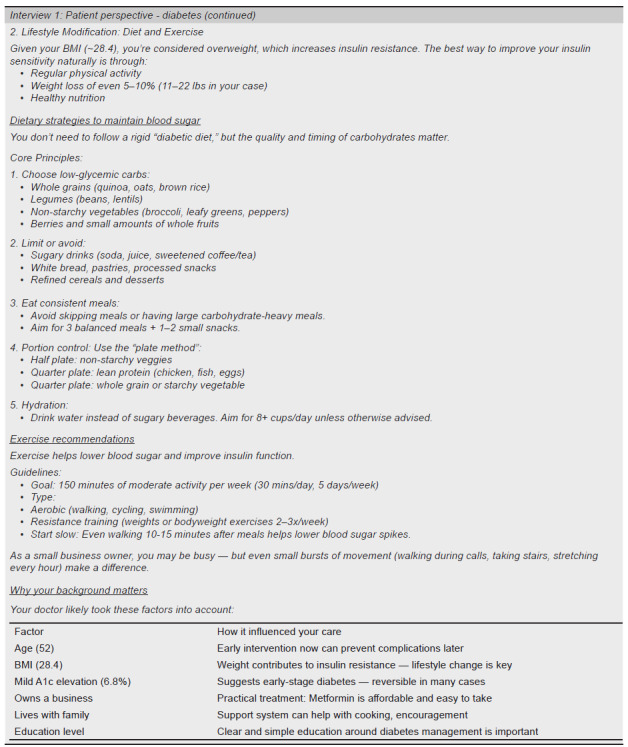

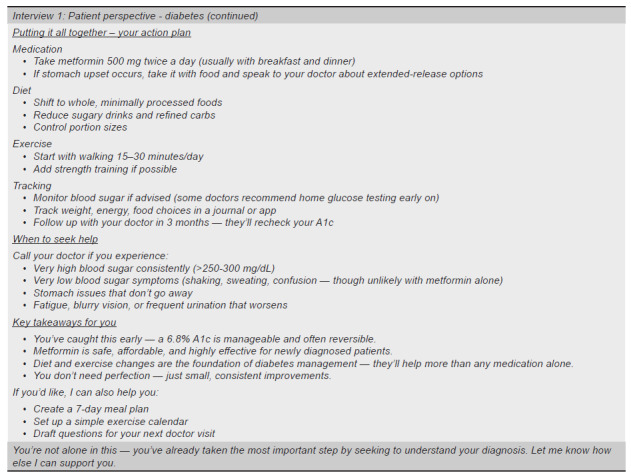
Chat GPT input prompt and generated output for a patient perspective regarding their diagnosis of type 2 diabetes (scenario 1). Output generated by GPT-4 on July 22, 2025, https://openai.com *BMI*, body mass index.

**Figure 5B:**
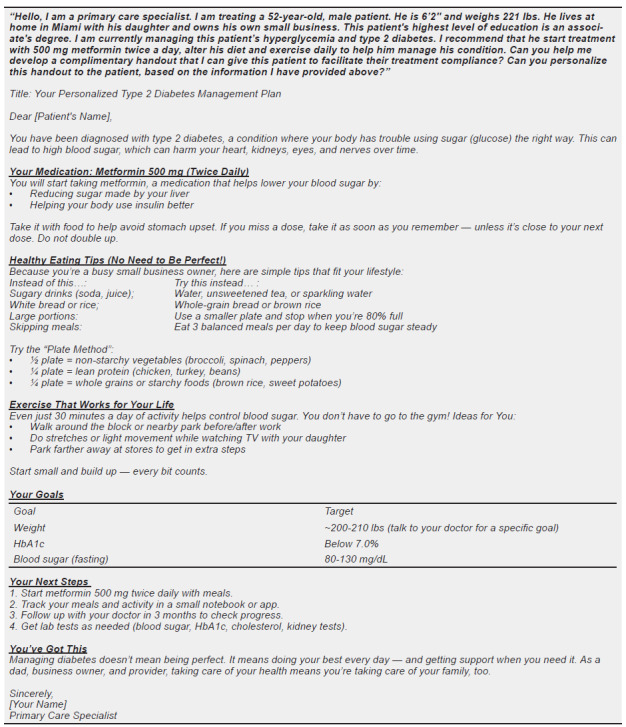
Chat GPT input prompt and generated output for a physician perspective regarding a care plan for their patient with a diagnosis of type 2 diabetes (scenario 2). Output generated by GPT-4 on 7/22/25, https://openai.com.

**Table:** Word count and readability via FKRE scores for each LLM output.

	Word count	FKRE score
Unstructured input prompt	415	51.4
Patient perspective – diabetes	948	49.8
Physician perspective – diabetes	408	79.8
Patient perspective - outpatient TJA	760	39.7
Physician perspective - outpatient TJA	528	62.6
Patient perspective - adolescent STI education	524	52.6
Physician perspective - adolescent STI education	383	65.7

*FKRE*, Flesch-Kincaid Reading Ease score (0–100 scale, higher scores are easier to read); *TJA*, Total Joint Arthroplasty; *STI*, sexually transmitted infection.

## References

[1] Eysenbach G (2023). The role of ChatGPT, large language models, and artificial intelligence in medical education: a conversation with ChatGPT and a call for papers. JMIR Med Educ.

[2] Laizure SC (2024). Caution: ChatGPT Doesn’t Know What You Are Asking and Doesn’t Know What It Is Saying. J Pediatr Pharmacol Ther.

[3] Andalib S, Solomon SS, Picton BG (2025). Source characteristics influence AI-enabled orthopaedic text simplification: recommendations for the future. JB JS Open Access.

[4] Spina A, Andalib S, Flores D (2024). Evaluation of large language models in personalizing medical information: instrument validation study. JMIR AI.

[5] Sallam M (2023). ChatGPT utility in healthcare education, research, and practice: systematic review on the promising perspectives and valid concerns. Healthcare (Basel).

[6] Gilson A, Safranek CW, Huang T (2023). How does ChatGPT perform on the United States Medical Licensing Examination? The implications of large language models for medical education and knowledge assessment. JMIR Med Educ.

[7] Gupta R, Park JB, Bisht C (2023). Expanding cosmetic plastic surgery research using ChatGPT. Aesthet Surg J.

[8] Yeo YH, Samaan JS, Ng WH (2023). Assessing the performance of ChatGPT in answering questions regarding cirrhosis and hepatocellular carcinoma. Clin Mol Hepatol.

[9] Ayers JW, Poliak A, Dredze M (2023). Comparing physician and artificial intelligence Chatbot responses to patient questions posted to a public social media forum. JAMA Intern Med.

[10] Nouha Dziri SM, Yu M, Zaiane O On the origin of hallucinations in conversational models: Is it the datasets or the nodels?.

[11] Andalib S, Spina A, Picton B (2025). Using AI to translate and simplify Spanish orthopedic medical text: instrument validation study. JMIR AI.

[12] Johnson SB, Parsons M, Dorff T (2022). Cancer misinformation and harmful information on Facebook and other social media: a brief report. J Natl Cancer Inst.

[13] Nolke L, Mensing M, Kramer A (2015). Sociodemographic and health-(care-)related characteristics of online health information seekers: a cross-sectional German study. BMC Public Health.

[14] Picton B, Andalib S, Spina A (2025). Assessing AI simplification of medical texts: readability and content fidelity. Int J Med Inform.

[15] Scherr R, Halaseh FF, Spina A (2023). ChatGPT interactive medical simulations for early clinical education: case study. JMIR Med Educ.

[16] Scherr R, Spina A, Dao A (2025). Novel evaluation metric and quantified performance of ChatGPT-4 patient management simulations for early clinical education: experimental study. JMIR Form Res.

[17] Spina AC, Fereydouni P, Tang JN (2025). Tailoring glaucoma education using large language models: addressing health disparities in patient comprehension. Medicine (Baltimore).

[18] Kirwan JP, Sacks J, Nieuwoudt S (2017). The essential role of exercise in the management of type 2 diabetes. Cleve Clin J Med.

[19] Halaseh FF, Yang JS, Danza CN (2024). ChatGPT’s role in improving education among patients seeking emergency medical treatment. West J Emerg Med.

[20] Yau JY, Saadat S, Hsu E (2024). Accuracy of prospective assessments of 4 large language model chatbot responses to patient questions about emergency care: experimental comparative study. J Med Internet Res.

[21] Reiners F, Sturm J, Bouw LJW (2019). Sociodemographic factors influencing the use of eHealth in people with chronic diseases. Int J Environ Res Public Health.

[22] Tonelli M, Wiebe N, Manns BJ (2018). Comparison of the complexity of patients seen by different medical subspecialists in a universal health care system. JAMA Netw Open.

[23] Centers for Disease Control and Prevention (2022). Type 2 Diabetes.

[24] Forouhi NG (2023). Embracing complexity: making sense of diet, nutrition, obesity and type 2 diabetes. Diabetologia.

[25] Nam S, Chesla C, Stotts NA (2011). Barriers to diabetes management: patient and provider factors. Diabetes Res Clin Pract.

[26] Chrvala CA, Sherr D, Lipman RD (2016). Diabetes self-management education for adults with type 2 diabetes mellitus: a systematic review of the effect on glycemic control. Patient Educ Couns.

[27] Powers MA, Bardsley J, Cypress M (2015). Diabetes self-management education and support in type 2 diabetes: a joint position statement of the American Diabetes Association, the American Association of Diabetes Educators, and the Academy of Nutrition and Dietetics. J Acad Nutr Diet.

[28] Tatulashvili S, Fagherazzi G, Dow C (2020). Socioeconomic inequalities and type 2 diabetes complications: a systematic review. Diabetes Metab.

