# Supplementary material for: An Organized Approach to Using Large Language Models for Medical Information
Source: West J Emerg Med. 2025 Dec 20;27(1):194–204. doi: 10.5811/westjem.46577 (PMC12815512; doi:10.5811/westjem.46577)
Supplement: Supplementary file 1 [file wjem-27-194-s001.docx]

| **Annotated Input Prompt 3: Patient Perspective - Outpatient TJA** |
| --- |
| “Hello, I am a 67-year-old female patient. I am 5’4” and I weigh 103 lbs. I am a retired teacher and live at home in Orange, California with my husband. The highest level of education I have received is a bachelor's degree. I came to the emergency department with a **hip fracture**. The orthopedic surgeon recommended that ***I have an open reduction with intramedullary fixation (ORIF with IMN)***. While he explained his rationale for ***the surgery to be done at an ambulatory surgical*** center, I cannot remember it and I am starting to get worried because I cannot get in contact with him to ask him questions at the moment. Can you explain to me my doctor’s recommendation, given the information I have provided you about myself. Can you make this response extremely comprehensive, in order to provide me with an in-depth understanding of what is going on.” |

**Supplemental Figure 1A** – Chat GPT Annotated Input Prompt for a Patient Perspective Regarding their Outpatient Total Joint Arthroplasty (TJA) (Scenario 3).

| **Annotated Input Prompt 4: Physician Perspective - Outpatient TJA** |
| --- |
| “Hello, I am an Orthopedic Surgeon that specializes in joint replacements who is planning to operate on a 67-year-old female patient that presented to the ER with a hip fracture. This patient is 5’4” and weighs 103 lbs. This patient is a retired teacher and lives at home in Orange, California with her husband. This patient's highest level of education is a bachelor’s degree. I will be performing an **open reduction with intramedullary fixation (ORIF with IMN).** I specifically recommend the patient have ***the surgery performed at an ambulatory surgical center*** and after a discussion about the pros and cons of ***outpatient hip replacement*** the patient agreed to my plan. I want to create an educational information sheet for this patient on ***how to best prepare for this surgery, what to expect from this surgery, especially with it taking place in an outpatient setting, and what to do post-operatiation in order to optimize her chance for a successful outcome***. Can you create a personalized informational sheet for this exact patient based on the information I have provided above?” |

**Supplemental Figure 1B** – Chat GPT Annotated Input Prompt for a Physician Perspective Regarding a Care Plan for their Patient Undergoing Outpatient Total Joint Arthroplasty (TJA) (Scenario 4).

| **Annotated Input Prompt 5: Patient Perspective - Adolescent STI Education** |
| --- |
| “Hello, I am a 17-year-old female patient who lives in Houston, Texas. I am 5’2” and weigh 109 lbs. I am a senior in high school and I live at home with my parents and two younger brothers. I do not currently have a job. I was recently diagnosed in the emergency department with **gonorrhea**. I was never taught much about STIs, and to be honest, ***I did not know there were STIs other than HIV/AIDS***. My pediatrician recommended treatment with ***ceftriaxone*** which I agreed to and underwent. Can you tell me more about STIs? What causes them and how can I prevent them? Can you personalize the explanation to me based on the information I provided above?” |

**Supplemental Figure 1C** – Chat GPT Annotated Input Prompt for an Adolescent Patient Perspective Regarding Sexually Transmitted Infection (STI) Education (Scenario 5).

| **Annotated Input Prompt 6: Physician Perspective - Adolescent STI Education** |
| --- |
| "Hello, I am a pediatrician working in Houston, Texas. I am working with a 17-year-old female patient who is a senior in high school. She is 5’2” and weighs 109 lbs. She lives at home with her two parents and younger brothers. She is not currently employed. She was recently diagnosed in the emergency department with **gonorrhea**. She has limited knowledge about ***STIs***. I have discussed ***STIs***, causes, and prevention with her, but I'd like to provide her with a comprehensive, personalized information sheet for her reference. Please help me create a one-page information sheet on STIs, focusing on causes, prevention, and **gonorrhea** specifically, tailored to her background and educational level." |

**Supplemental Figure 1D** - Chat GPT Annotated Input Prompt for a Physician Perspective Regarding their Adolescent Patient’s Sexually Transmitted Infection (STI) Diagnosis. (Scenario 6).

| **Interview 3: Patient Perspective - Outpatient TJA** |
| --- |
| **“Hello, I am a 67-year-old female patient. I am 5’4” and I weigh 103 lbs. I am a retired teacher and live at home in Orange, California with my husband. The highest level of education I have received is a bachelor's degree. I came to the emergency department with a hip fracture. The orthopedic surgeon recommended that I have an open reduction with intramedullary fixation (ORIF with IMN). While he explained his rationale for the surgery to be done at an ambulatory surgical center, I cannot remember it and I am starting to get worried because I cannot get in contact with him to ask him questions at the moment. Can you explain to me my doctor’s recommendation, given the information I have provided you about myself. Can you make this response extremely comprehensive, in order to provide me with an in-depth understanding of what is going on.”**  *Absolutely. I’m sorry you’re going through this, and I hope I can provide you with the clarity and reassurance you need. Based on what you’ve told* *me, here is a comprehensive explanation of your situation and your doctor’s recommendation:*    *Your Diagnosis: Hip Fracture*    *A hip fracture refers to a break in the upper portion of your femur (thigh bone), near the hip joint. In older adults, hip fractures often happen after a fall, and they’re more common in women due to age-related bone loss (osteoporosis).*    *Your height and weight (5’4”, 103 lbs) suggest that you have a slender frame, which may contribute to increased bone fragility. Being 67 years old also increases your risk, as bone density naturally declines with age.*      *The Recommended Procedure: ORIF with Intramedullary Nail (IMN)*    *ORIF stands for Open Reduction and Internal Fixation, and it’s a surgical procedure used to treat fractures that are displaced or unstable. Here’s what each part means:*   - *Open Reduction: The orthopedic surgeon makes an incision to expose the broken bone and physically re-align it into its proper position.* - *Internal Fixation: The realigned bone fragments are then held together using metal hardware—in your case, an intramedullary nail (IMN), which is a metal rod inserted into the marrow canal of the femur.*     *Why an Intramedullary Nail?*    *An IMN is often the best choice for certain types of femoral fractures, especially intertrochanteric or subtrochanteric fractures, which are common in older adults. This technique provides:*   - *Strong internal support* - *Faster healing and mobilization* - *Lower risk of complications compared to plates/screws alone*     *Why an Ambulatory Surgical Center (ASC)?*    *An ambulatory surgical center is a facility designed to provide same-day surgeries that don’t require overnight hospital stays. Here’s why your surgeon might have recommended an ASC:*    *Medical Stability*    *Your overall profile—no mention of chronic illnesses, normal body weight, non-obese, and likely good general health for your age—suggests you’re medically stable enough to undergo surgery without the need for prolonged post-op monitoring.*    *Lower Risk of Complications*   - *You are not immunocompromised.* - *You don’t have risk factors like morbid obesity, severe heart disease, or uncontrolled diabetes.* - *Your relatively young age for this type of fracture (many occur in patients >75) and your active, independent lifestyle suggest a lower risk of post-operative complications.*     *Reduced Exposure to Hospital-Acquired Infections*    *Hospitals—**especially for elderly patients—can pose risks such as pneumonia, urinary tract infections, and hospital-acquired infections. ASCs have lower infection rates overall.*    *Faster Recovery and Discharge*    *Many ASCs are streamlined for orthopedic cases and have:*   - *Efficient protocols for regional anesthesia* - *Early physical therapy initiation* - *Discharge planning within hours of surgery*     *Cost Savings*    *Surgery at an ASC is usually much less expensive than hospital-based care. As a retired person on a fixed income, this could be a consideration your doctor made on your behalf.*    *Will I Be Safe?*    *Yes—if the ASC was recommended after a careful medical evaluation, it means your surgeon believes:*   - *Your fracture type is suitable for a same-day repair.* - *You have no major medical conditions that require inpatient monitoring.* - *You have a supportive home environment (you live with your husband, which helps ensure you won’t be alone during recovery).* - *Postoperative care like physical therapy and follow-ups can be safely managed on an outpatient basis.*     *What Should You Do Next?*    *If you’re still feeling uncertain, here’s what you can do:*   - *Contact the surgical scheduler or orthopedic clinic nurse — they often have access to your chart and may be able to explain the decision further.* - *Ask for a copy of the surgical plan or your imaging — this can help another provider confirm the appropriateness of the procedure.* - *Reach out to your primary care physician (PCP) — they may be able to review the recommendation with you and ensure it aligns with your overall health status.*     *Prepare for your recovery at home:*   - *Arrange a walker or cane if needed* - *Have someone available to help for a few days post-op* - *Plan meals and medications in advance*     *Summary*    *Your orthopedic surgeon has recommended ORIF with IMN to fix your hip fracture—this is a well-established, evidence-based approach, especially in healthy, mobile older adults like yourself. The use of an ambulatory surgical center likely reflects your low risk of complications, your stable health status, and the benefits of faster recovery and lower cost.*    *You’re not alone in this—many patients like you undergo the same procedure safely and successfully in ASCs. If you have further concerns, try to connect with the orthopedic team, or your primary care doctor, and ask about getting a pre-op consultation or second opinion.* |

**Supplemental Figure 2A** – Chat GPT Input Prompt and Generated Output for a Patient Perspective Regarding their Outpatient Total Joint Arthroplasty (TJA) (Scenario 3). Output generated by GPT-4 on 7/22/25, [https://openai.com](https://openai.com/).

| **Interview 4: Physician Perspective - Outpatient TJA** |
| --- |
| **“Hello, I am an Orthopedic Surgeon that specializes in joint replacements who is planning to operate on a 67-year-old female patient that presented to the ER with a hip fracture. This patient is 5’4” and weighs 103 lbs. This patient is a retired teacher and lives at home in Orange, California with her husband. This patient's highest level of education is a bachelor’s degree. I will be performing an open reduction with intramedullary fixation (ORIF with IMN). I specifically recommend the patient have the surgery performed at an ambulatory surgical center and after a discussion about the pros and cons of outpatient hip replacement the patient agreed to my plan. I want to create an educational information sheet for this patient on how to best prepare for this surgery, what to expect from this surgery, especially with it taking place in an outpatient setting, and what to do post-operatiation in order to optimize her chance for a successful outcome. Can you create a personalized informational sheet for this exact patient based on the information I have provided above?”**  *Title: Preparing for Your Outpatient Total Hip Replacement Surgery*  *Dear [Patient's Name],*  *You’ve experienced a hip fracture, which is common in older adults. An open reduction with intramedullary fixation (ORIF with IMN) helps realign and stabilize the broken bone using a metal rod placed in the center of the bone (intramedullary nail).*  *We are performing your surgery at an ambulatory surgical center (ASC), which offers:*   - *Faster recovery in the comfort of your home* - *Lower risk of hospital-acquired infections* - *More personalized care in a focused setting* - *Shorter overall stay (often discharged the same day)*     *You and your surgeon discussed the benefits and felt this setting is right for you.*      *What to Do Before Surgery*    *1. Medical Clearance*  *You may need pre-operative testing (labs, EKG, chest X-ray) and a checkup by your primary care provider.*    *2. Medication Review*  *Tell us about all medications you take. We may adjust:*   - *Blood thinners (e.g., aspirin, Plavix)* - *Diabetes meds (e.g., metformin, insulin)* - *Supplements or herbal products*     *3. Plan for Home Support*  *Since you’ll go home the same day, arrange:*   - *A responsible adult to drive you home and stay the first 24–48 hours* - *A walker or cane available if needed* - *A safe environment (remove tripping hazards, place needed items at waist level)*     *4. Fasting Instructions*  *No food or drink after midnight before your surgery unless told otherwise.*    *What to Expect on Surgery Day*    *Arrival at the ASC*   - *You’ll check in early, change into a surgical gown, and meet your care team.* - *Anesthesia will be discussed – likely spinal or general anesthesia.*     *The Procedure*   - *Duration: Typically 1 to 2 hours* - *A small incision is made to align the fracture and insert the nail.* - *You’ll spend time in the recovery area before going home the same day.*     *After Surgery: Recovery at Home*    *Pain Management*   - *You’ll receive medications to control pain.* - *Take them as directed; notify us if pain feels unmanageable.*     *Mobility & Safety*   - *You’ll start moving with assistance the same day.* - *Use your walker or crutches as instructed.* - *Avoid putting full weight on the leg unless your surgeon says it’s okay.*     *Wound Care*   - *Keep the incision clean and dry.* - *Watch for signs of infection (redness, warmth, pus, fever).*     *Follow-Up*   - *You’ll return for a follow-up appointment in 1–2 weeks.* - *X-rays may be done to check healing.*     *Physical Therapy*   - *You may begin PT within a few days to regain strength and mobility.* - *Stick to your therapy plan—it’s key to a successful recovery.*     *When to Call Us*    *Call your surgeon if you notice:*   - *Fever >101°F* - *Increased pain, swelling, or redness around the incision* - *Drainage or foul odor from the wound* - *Numbness or inability to move your leg* - *Difficulty breathing or chest pain (call 911 immediately)*     *Your Checklist*     - *Arrange transportation and home help* - *Stop or adjust meds as instructed* - *Set up home safety (**walker, remove rugs, etc.)* - *Pack a small bag with ID, meds list, and comfortable clothes* - *Follow pre-surgery fasting instructions*     *Your Recovery Goals*  *We want to help you return to your regular activities, maintain your independence, and avoid future complications. Your role in preparing, following post-op instructions, and participating in rehab is vital.*    *Thank you for trusting us with your care!*  *Sincerely,*  *[Your Name, MD]*  *Orthopedic Surgeon* |

**Supplemental Figure 2B** – Chat GPT Input Prompt and Generated Output Physician Perspective Regarding a Care Plan for their Patient Undergoing Outpatient Total Joint Arthroplasty (TJA) (Scenario 4). Output generated by GPT-4 on 5/1/23, [https://openai.com](https://openai.com/).

| **Interview 5: Patient Perspective - Adolescent STI Education** |
| --- |
| **“Hello, I am a 17-year-old female patient who lives in Houston, Texas. I am 5’2” and weigh 109 lbs. I am a senior in high school and I live at home with my parents and two younger brothers. I do not currently have a job. I was recently diagnosed in the emergency department with gonorrhea. I was never taught much about STIs, and to be honest, I did not know there were STIs other than HIV/AIDS. My pediatrician recommended treatment with ceftriaxone which I agreed to and underwent. Can you tell me more about STIs? What causes them and how can I prevent them? Can you personalize the explanation to me based on the information I provided above?”**  *Absolutely—and thank you for your honesty and courage in seeking more information. It’s incredibly important to understand STIs (sexually transmitted infections), and you are not alone—many people your age haven’t been taught much about them either.*    *What Are STIs?*    *STIs are infections passed from one person to another during sexual activity—this can include vaginal, oral, or anal sex, and sometimes even close skin-to-skin contact.*    *There are many STIs, not just HIV. Some of the most common include:*   - *Gonorrhea (what you were recently diagnosed with)* - *Chlamydia* - *Human papillomavirus (HPV)* - *Herpes (HSV)* - *Syphilis* - *Trichomoniasis* - *HIV/AIDS*     *What Causes STIs?*    *STIs are caused by different types of germs:*   - *Bacteria (like gonorrhea, chlamydia, syphilis) — often curable with antibiotics* - *Viruses (like HPV, herpes, HIV) — not curable, but manageable with medication* - *Parasites (like trichomoniasis) — treatable with specific medicine*     *Why Gonorrhea Was Treated with Ceftriaxone*    *You received ceftriaxone, a strong antibiotic given by injection, which is the recommended treatment for gonorrhea. Gonorrhea is caused by a bacteria (Neisseria gonorrhoeae), and ceftriaxone kills this bacteria.*    *It’s important to follow up with your doctor if you still have symptoms after treatment or if you’re worried about other possible infections.*    *How Can You Protect Yourself? (Personalized to You)*    *You’re a 17-year-old high school student just starting to understand sexual health. Here are some tips that apply specifically to your age and life:*   1. *Education is Power: It’s great you’re asking questions—keep learning. Schools often don’t teach everything, but you can speak with your pediatrician or find reliable online resources like the CDC or Planned Parenthood.* 2. *Condoms Help Prevent STIs: Male and female condoms are very effective at preventing many STIs, including gonorrhea, chlamydia, and HIV. Even if you’re using other birth control (like the pill), condoms are still needed to protect against infections.* 3. *Get Regular STI Testing: If you’re sexually active, routine testing is important—even if you feel fine. Some STIs (like chlamydia or HPV) may not cause symptoms but can still cause long-term problems like infertility.* 4. *Limit Risky Sexual Behaviors: The fewer partners you have and the more open communication you have about testing and protection, the lower your risk. Avoid sex when under the influence of drugs or alcohol, as it can make it harder to make safe decisions.* 5. *Get the HPV Vaccine: If you haven’t already, ask your doctor about the HPV vaccine. It protects against certain strains of HPV that can cause cervical cancer and genital warts.* 6. *Talk to Trusted Adults and Providers: Your pediatrician is there to help you, not judge you. They can talk privately with you and help you stay healthy.*     *When to See a Doctor*    *Always seek care if you have:*   - *Unusual vaginal discharge* - *Burning or pain during urination* - *Sores or bumps on your genitals* - *Lower belly or pelvic pain* - *Bleeding between periods or after sex*     *You’re doing the right thing by getting treated and asking questions. Now is a great time to take charge of your health. You deserve to feel safe, supported, and informed. If you ever feel unsure about something, know that doctors, school nurses, and even certain clinics like Planned Parenthood are there to support you confidentially.* |

**Supplemental Figure 2C** – Chat GPT Input Prompt and Generated Output for an Adolescent Patient Perspective Regarding Sexually Transmitted Infection (STI) Education (Scenario 5). Output generated by GPT-4 on 5/1/23, [https://openai.com](https://openai.com/).

| **Interview 6: Physician Perspective - Adolescent STI Education** |
| --- |
| **"Hello, I am a pediatrician working in Houston, Texas. I am working with a 17-year-old female patient who is a senior in high school. She is 5’2” and weighs 109 lbs. She lives at home with her two parents and younger brothers. She is not currently employed. She was recently diagnosed in the emergency department with gonorrhea. She has limited knowledge about STIs. I have discussed STIs, causes, and prevention with her, but I'd like to provide her with a comprehensive, personalized information sheet for her reference. Please help me create a one-page information sheet on STIs, focusing on causes, prevention, and gonorrhea specifically, tailored to her background and educational level."**  *Title: STIs: Causes, Prevention, and Gonorrhea Information Sheet*  *What Are STIs?*    *STIs (Sexually Transmitted Infections) are infections passed from one person to another through sexual contact. They can affect anyone—regardless of age, background, or relationship status.*    *You may have heard of HIV or AIDS, but there are many other STIs too—like chlamydia, gonorrhea, herpes, HPV, syphilis, and trichomoniasis. Some STIs can cause symptoms, while others can stay “silent” in your body.*    *What Causes STIs?*    *STIs are caused by bacteria, viruses, or parasites that spread during:*   - *Vaginal, anal, or oral sex* - *Skin-to-skin contact (in some cases)* - *Sharing contaminated needles* - *From mother to baby during birth*     *You cannot get an STI from hugging, sharing a toilet, or eating food someone else touched.*    *Gonorrhea: What You Need to Know*    *You were recently diagnosed with gonorrhea, a common STI caused by bacteria.*  *Here’s what you should know:*   - *It often doesn’t cause symptoms, especially in females* - *If it does, you may notice pain when peeing, vaginal discharge, or lower belly pain* - *Untreated gonorrhea can lead to serious issues like infertility or pelvic inflammatory disease* - *The good news? It’s curable with antibiotics, like the ceftriaxone you were given*     *It’s important to:*   - *Finish all medications* - *Avoid sex for 7 days after treatment* - *Tell your partner(s) so they can get tested and treated too* - *Get retested in 3 months to make sure you’re in the clear*     *How Can You Prevent STIs?*    *Here are ways to protect yourself:*     - *Use condoms every time you have sex – they reduce the risk of most STIs* - *Limit the number of sexual partners* - *Get tested regularly, even if you feel fine* - *Talk openly with any partner about STIs before sex* - *Avoid drugs/alcohol that impair judgment in sexual situations*     *Abstinence (not having sex) is the only 100% way to prevent STIs, but if you’re sexually active, being informed and cautious helps protect your health.*    *You’re Not Alone*    *It’s okay to have questions. STIs are common and treatable, and asking questions shows you’re taking care of your health. If you ever feel unsure or worried, your doctor is here for you — no judgment.*    *If you’d like, here are some trustworthy websites:*   - *TeensHealth.org* - *CDC: STDs* - *Planned Parenthood*     *You’ve Got This.*  *Taking the first step toward understanding your health is a powerful thing. You’re smart, capable, and supported.* |

**Supplemental Figure 2D** – Chat GPT Input Prompt and Generated Output for a Physician Perspective Regarding their Adolescent Patient’s Sexually Transmitted Infection (STI) Diagnosis. (Scenario 6). Output generated by GPT-4 on 5/1/23, <https://openai.com>.
